# Excipient Nanoemulsions for Improving Oral Bioavailability of Bioactives

**DOI:** 10.3390/nano6010017

**Published:** 2016-01-14

**Authors:** Laura Salvia-Trujillo, Olga Martín-Belloso, David Julian McClements

**Affiliations:** 1 Department of Food Science, University of Massachusetts, Amherst, MA 01003, USA; laura@foodsci.umass.edu; 2 Department of Food Technology, University of Lleida, Lleida 25193, Spain; omartin@tecal.udl.cat; 3 Department of Biochemistry, Faculty of Science, King Abdulaziz University, P.O. Box 80203, Jeddah 21589, Saudi Arabia

**Keywords:** excipient, nanoemulsions, bioactive, delivery, functional, oral bioavailability

## Abstract

The oral bioavailability of many hydrophobic bioactive compounds found in natural food products (such as vitamins and nutraceuticals in fruits and vegetables) is relatively low due to their low bioaccessibility, chemical instability, or poor absorption. Most previous research has therefore focused on the design of delivery systems to incorporate isolated bioactive compounds into food products. However, a more sustainable and cost-effect approach to enhancing the functionality of bioactive compounds is to leave them within their natural environment, but specifically design excipient foods that enhance their bioavailability. *Excipient foods* typically do not have functionality themselves but they have the capacity to enhance the functionality of nutrients present in natural foods by altering their bioaccessibility, absorption, and/or chemical transformation. In this review article we present the use of excipient nanoemulsions for increasing the bioavailability of bioactive components from fruits and vegetables. Nanoemulsions present several advantages over other food systems for this application, such as the ability to incorporate hydrophilic, amphiphilic, and lipophilic excipient ingredients, high physical stability, and rapid gastrointestinal digestibility. The design, fabrication, and application of nanoemulsions as excipient foods will therefore be described in this article.

## 1. Introduction

### 1.1. Concept of Excipient Foods

The functionality of bioactive compounds present in foods, dietary supplements, or drugs largely depends on their fate in the gastrointestinal tract (GIT). The bioavailability of many health-promoting compounds is generally low due to their low bioaccessibility, susceptibility to degradation, or poor absorption profile. The processes that govern the oral bioavailability of bioactive compounds are complex and include liberation of the bioactive from the ingested drug, supplement, or food [1]; solubilization within gastrointestinal fluids [2,3]; transport into or out of epithelial cells [4,5]; and/or, biochemical or chemical transformations [6,7]. In this article, we focus on enhancing the bioavailability of lipophilic bioactive compounds found in foods (particularly fruits and vegetables), but the same principles could also be applied to supplements and pharmaceuticals. There is strong evidence that the nature of foods influences the bioavailability of co-ingested bioactive compounds, thereby altering their bioactivity. The ingredients present in the food matrix might favor or inhibit the liberation and solubilization of functional compounds during digestion [8,9]. Therefore, there is an opportunity to optimize the formulation of food products to improve the bioavailability of bioactive compounds. There are several strategies that can be used to enhance the liberation, solubilization and absorption of bioactive compounds. Colloidal delivery systems can be used to incorporate isolated lipophilic bioactive ingredients into food products, such as nanoemulsions, emulsions, or solid lipid nanoparticles [10,11]. In this case, the bioactive compound is first extracted from a natural source and it is then solubilized in a lipid phase prior to the formation of the colloidal delivery system that will be ultimately incorporated in a food product. Alternatively, the bioactive compounds might be left in their natural source (fruit or vegetable) and consumed along with a specific formulation able to boost their bioactivity. In this context, the concept of excipient foods has been introduced as foods that are specifically designed to improve the bioavailability of bioactive components [12]. Excipient foods might or might not have an intrinsic bioactivity themselves, but when they co-ingested with other foods they can boost the bioactivity of any bioactive compounds present.

### 1.2. Nanoemulsions as Excipient Foods

Nanoemulsions have been described as excellent carriers for lipophilic bioactive compounds with enhanced properties compared to conventional emulsions. Typically, nanoemulsions are defined as oil-in-water emulsions with a very small droplet size (*r* < 100 nm). Their small droplet dimensions confer them with unique properties, such as improved physical stability, high optical clarity, and enhanced bioavailability [13]. Due to their small droplet size, nanoemulsions have a large surface area and can therefore interact strongly with biological components in the GIT. For instance, nanoemulsions typically have a higher digestion rate in the gastrointestinal tract compared to conventional emulsions since they have more binding sites available for digestive enzymes, such as lipase [14]. Moreover, the small droplet size may favor the rapid transfer of naturally occurring hydrophobic bioactives present in foods into the oil droplets. Thus, in this review article, we focus on describing the use of nanoemulsions as excipient foods and how their composition and structure can be modulated to achieve an optimal efficacy of the functional components of natural food products.

## 2. Excipient Foods

**Figure 1:**
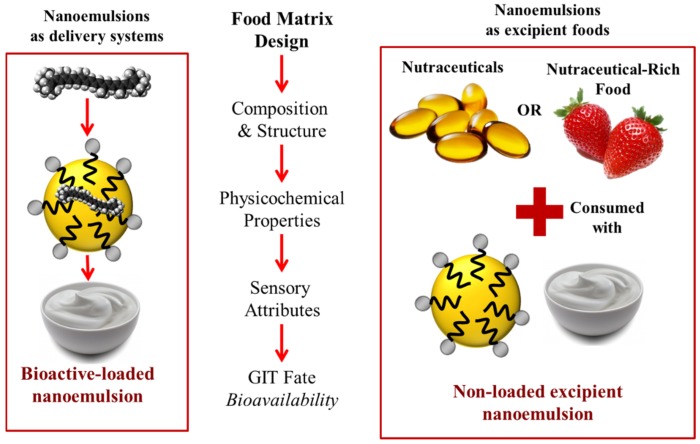
Schematic diagram of the difference between integrated and non-integrated excipient foods. For integrated excipient foods the bioactive component (pharmaceutical or nutraceutical) is dispersed within the excipient food matrix, but for non-integrated excipient foods the bioactive component is in another product that is co-ingested with the excipient food.

The relationship between the composition of foods and many health issues is well documented and consumers are nowadays generally aware of the importance of a balanced diet. However, the lifestyle of many modern consumers makes it difficult to ingest all of the nutrients needed to maintain normal body functions or promote good health. The use of food products or nutraceuticals to treat health conditions has already been described and is intended to prevent or cure certain diseases. Medical or functional foods fall in the category of foods that are specifically designed to ameliorate human health problems [15]. For instance, a *medical food* is defined by the Food and Drug Administration (FDA) as “*a food which is formulated to be consumed or administered entirely under the supervision of a physician and which is intended for the specific dietary management of a disease or condition for which distinctive nutritional requirements, based on recognized scientific principles, are established by medical evaluation*” [15]. On the other hand, *functional foods* are described as natural or processed foods that have been specifically been fortified with bioactive compounds. Various types of functional food products already exist in the market, such as milks fortified with vitamin D, orange juices enriched with calcium, yogurts containing probiotics, spreads fortified with phytosterols, and breakfast cereals containing ω-3 fatty acids, vitamins, and minerals [16]. Nevertheless, the concept of designing healthier food products has recently gone a step further to optimize the promotion of the intrinsic health properties of natural food products. In this context, *excipient foods* have been introduced as foods that are able to improve the bioactivity of foods co-ingested with them [12] (Figure 1). Thus, excipient foods do not necessarily have bioactive properties but when ingested along with natural or processed food products they are able to promote the release and absorption of naturally occurring bioactive compounds. Excipient foods can be classified into two groups: (i) integrated excipient foods—the bioactive agents are dispersed into the excipient food formulation; (ii) non-integrated excipient foods—a bioactive-rich food is co-ingested with an excipient food formulation (Figure 1).

Conceptually, many types of foods products could potentially be designed to act as excipient foods. The type of product will be determined partly by the compatibility between the natural food and the excipient formulation. In Table 1 a number of examples are given for potential combinations of natural foods with high bioactive potential and excipient foods. For instance, an excipient salad dressing could be designed to enhance the bioavailability of carotenoids in salad vegetables. Indeed, there is already evidence that adding a certain amount of fat to a salad can increase the bioavailability of carotenoids [17]. Alternatively, an excipient sauce could be added during the cooking of vegetables, meat or fish. These excipient dressings or sauces may contain various food components that increase the bioavailability of the natural bioactive compounds present in foods, such as lipids that increase GIT solubility, antioxidants that inhibit chemical transformations, enzyme inhibitors that retard metabolism, or permeation enhancers that increase absorption.

**Table 1:** Examples of potential excipient foods to be co-ingested with natural food products to enhance the oral bioavailability of bioactive compounds.

Food Source	Compatible Excipient Food	Bioactive Compound
Salad	Salad dressing	Carotenoids
Fruits and berries	Cream, ice cream, yogurt	Flavonoids, vitamins, coenzyme Q10
Vegetables	Edible coatings, sauce	Carotenoids, vitamins, phytosterols/stanols
Nuts and seeds	Edible coatings, sauce	Flavonoids, vitamins
Meat	Sauce	Conjugated linoleic acids
Dairy products (cheese)	Sauce	Conjugated linoleic acids
Fish	Sauce	ω-3 fatty acids

## 3. Factors Limiting the Bioavailability of Bioactive Compounds

There are several factors that limit the oral bioavailability of bioactive compounds in foods. There is a need to better understand the fate of bioactive compounds during their passage through the GIT in order to formulate optimal excipient foods to enhance their oral bioavailability. An integrated approach has been recently described to explain the main factors limiting the oral bioavailability of bioactive compounds (Equation (1)) [18]:

BA = B* × A* × T*(1)

**Figure 2:**
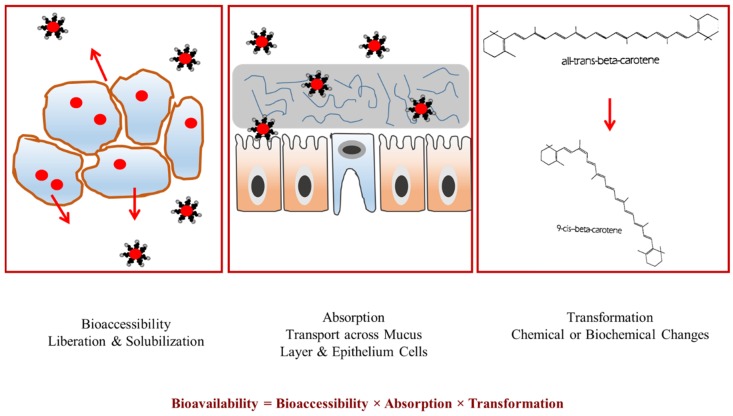
The overall oral bioavailability of bioactives is governed by three main factors: bioaccessibility; absorption; and transformation.

Here, BA is the oral bioavailability of a certain bioactive compound, B* is the bioaccessibility, A* is the absorption and T* is the molecular transformation. Thus, in order to maximize the oral bioavailability of a determined molecule, one has to enhance the fraction that will be bioaccessible, absorbed and in an active state after any changes in the molecular structure that might have occurred during digestion (Figure 2).

The classification of bioactive compounds according to the factors limiting their oral bioavailability has been recently been described using a nutraceutical bioavailability classification scheme (*NuBACS*) [9]. In this classification scheme, the many factors that limit the bioaccessibility, absorption, or transformation of a bioactive compound are indicated. Each compound falls into a different category depending on their behavior under GIT conditions, as well as their intrinsic properties. The bioaccessibility is mainly influenced by the liberation of the compound from the food matrix (L); poor solubility in the gastrointestinal fluids (S) and possible interactions that might promote insolubility (I). The main hurdles influencing the absorption are poor transport through the mucus layer (ML), tight junctions (TJ) or bilayer membranes (MP), inhibition of active transporters (AT), or the presence of efflux transporters (ET) in the epithelium cells. The transformation of bioactive compounds into more or less active forms is mainly affected by changes in their activity due to chemical (C) or metabolic (M) mechanisms.

The above-mentioned factors affecting the bioavailability have been summarized in Table 2. Moreover, the mechanism of action of how nanoemulsions might overcome the natural barriers that limit the oral bioavailability of bioactive compounds present in foods is explained later on in the review article.

**Table 2:** Examples of potential excipient foods to be co-ingested with natural food products to enhance the oral bioavailability of bioactive compounds.

Bioaccessibility	Absorption	Transformation
*Liberation*: the bioactive must be released from the drug, supplement of food matrix	*Mucus layer:* the bioactive must be transported across the mucus layer that coats epithelium cells	*Chemical transformation*: some bioactives undergo chemical degradation within the GIT
*Solubilization*: the bioactive must be solubilized within GIT fluids	*Tight junctions:* some bioactives may pass through tight junctions separating epithelium cells	*Biochemical transformation*: some bioactives are digested or metabolized by enzymes in the GIT
*Interactions*: the bioactive may interact with other food components	*Membrane permeation:* some bioactives may be transported through the phospholipid bilayer	
	*Active Transport:* some bioactives are transported by active transport proteins	
	*Efflux:* some bioactives are removed from epithelium cells by efflux proteins	

## 4. Nanoemulsions

### 4.1. Formation

The formation of food-grade nanoemulsions can be divided into high-energy or low-energy methods [13,19]. High-energy approaches utilize mechanical forces to intermingle oil and aqueous phases and to produce small droplets, and they include high-pressure homogenization, microfluidization, and sonication [20]. Sonicators generate high intensity ultrasonic waves that break up the oil and water phases into small droplets mainly through cavitation effects [21]. High-pressure homogenizers and microfluidizers also generate intense disruptive forces that can break oil droplets down to the sub-micron range [22]. Low-energy methods utilize changes in the composition or environment of a surfactant-oil-water system to spontaneously form small droplets, and they include spontaneous emulsification or phase-inversion methods [23]. Low-energy methods are able to produce nanoemulsions with simple equipment and avoid the temperature increase that is caused when using many high-energy approaches, with similar droplet size and stability characteristics. However, high surfactant-to-oil ratios (SOR) are needed (SOR > 0.7) when using low-energy methods to obtain small oil droplets [24], which might be a drawback to produce excipient foods containing a minimum amount of synthetic ingredients. In fact, the excess of surfactant used to produce nanoemulsions by low-energy methods might form micelles and cause destabilization phenomena in food products containing larger fat droplets [25]. Therefore, it is important to select the optimal fabrication method to obtain excipient nanoemulsions with optimal properties suitable for each type of food product that they will be co-ingested with. In this review article, we refer to both nanoemulsions produced by high-energy or low-energy methods.

### 4.2. Characteristics

**Figure 3:**
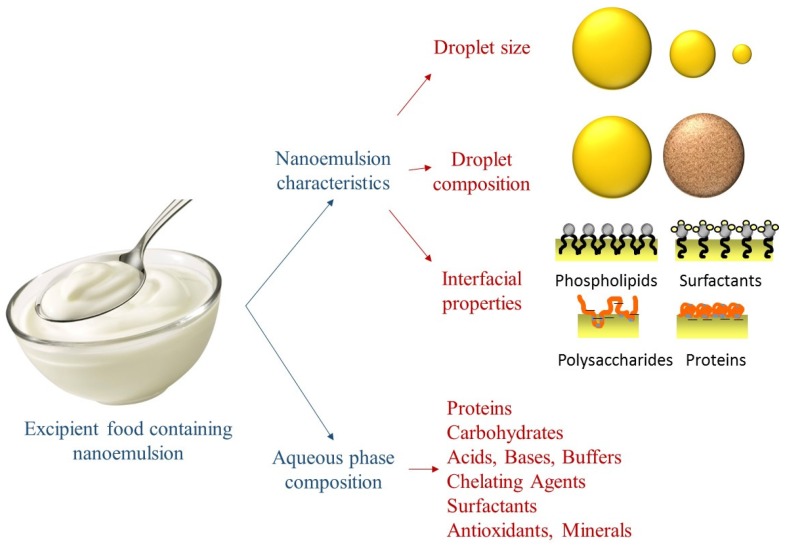
Schematic representation of the variables that can be modulated in order to formulate excipient nanoemulsions to enhance the oral bioavailability of naturally-occurring bioactive compounds.

The physicochemical characteristics and composition of nanoemulsions may be modulated in order to form excipient emulsions suitable for each target application (Figure 3) [26].

#### 4.2.1. Droplet Size

By definition the oil droplet radius in nanoemulsions is typically below 100 nm, which leads to properties significantly different from conventional emulsions [13]. The formation conditions, such as homogenization pressure and cycles, can be controlled to obtain different droplet sizes. Moreover, the emulsifier type and oil phase composition used will also determine the droplet size. Nanoemulsions containing smaller droplet sizes have been shown to be digested by lipases more rapidly than those containing larger droplet sizes, which is attributed to a higher surface area and therefore more binding sites for intestinal lipases [27]. Nanoemulsions with a faster digestibility might be used for bioactive compounds with a faster absorption pattern, where the presence of mixed micelles is required at early stages of the intestinal tract; whereas excipient nanoemulsions with a larger droplet size and therefore a slower digestion rate may be preferable to improve the absorption of bioactive compounds in foods that require a longer residence time in the GIT.

#### 4.2.2. Droplet Composition

Besides the intrinsic nutritional aspects of different types of oils and fats, the composition of oil droplets will determine the rate and extent of oil digestibility, as well as the structure of the mixed micelles formed after lipid digestion. Therefore, it is an important factor to take into account when formulating excipient nanoemulsions. For instance, nanoemulsions can be formulated with triacylglycerol oils, flavor oils, essential oils, mineral oils, or waxes. Oils with different fatty acid compositions have been shown to give significantly different lipid digestion rates, as well as leading to mixed micelle phases with appreciably different solubilization capacities for hydrophobic bioactives. Long chain triglycerides have shown to be digested more slowly than medium or short chain triglycerides, however, they lead to a higher bioaccessibility of carotenoids [28]. This fact was attributed to the fact that longer chain free fatty acids lead to the generation of mixed micelles with larger hydrophobic domains after digestion, which are able to incorporate hydrophobic bioactive compounds with large molecular dimensions, such as carotenoids [29]. This same principle might be applied to excipient nanoemulsions, whose oil phase composition can be specifically selected for each individual bioactive compound.

#### 4.2.3. Interfacial Properties

To form nanoemulsions, the use emulsifiers or surface-active molecules are required to stabilize the small oil droplets in the aqueous phase. Typically, emulsifiers adsorb at the oil-in-water interface thus conferring specific interfacial properties to the droplets. The oil-water interfacial composition, thickness, structure, or charge can be modulated using different types of emulsifiers to prepare the nanoemulsion, or by post-homogenization methods (such as emulsifier displacement or layer-by-layer deposition) [15]. The electrical charge of oil droplets determines their electrostatic interactions with other oil droplets or with other charged food compounds. Consequently, it influences the aggregation state of the lipid droplets (stable, flocculated or coalesced), which alters the exposed surface area where digestive enzymes adsorb. Moreover, the nature of the interface will influence the ability of bile salts and digestive enzymes to adsorb to the lipid droplet surfaces and initiate lipolysis [30]. Moreover, the surfactants and emulsifiers used to formulate nanoemulsions may also have an impact on the solubilization capacity of bioactive compounds in the intestinal juices. Consequently, the selection of an appropriate emulsifier must be optimized to ensure good bioaccessibility.

### 4.3. Mechanisms of Action

The design of nanoemulsions can be optimized to enhance the oral bioavailability of bioactive compounds in the GIT altering their compositions or structures. In this section, we highlight how discuss excipient nanoemulsions can be designed to alter the bioaccessibility, absorption, or transformation of bioactive compounds.

#### 4.3.1. Bioaccessibility

The bioaccessibility is defined as the amount of a bioactive agent that is present within the gastrointestinal tract in a form suitable for absorption (*m*_B_) compared to the total amount ingested (*m*_Total_) (Equation (2)):

B* = 100 × *m*_B_/*m*_Total_ (2)

In the case of lipophilic bioactive compounds, the fraction of bioactive compound that is solubilized in the micelle phase after the small intestine phase is considered to be potentially absorbable. There are several strategies that can be used to increase bioaccessibility by formulating nanoemulsions, mainly divided in efforts towards a higher release from the food matrix or a higher solubilization in the intestinal juices.

##### Changes in the Bioactive Release from the Food Matrix

Depending on the type of food product, bioactives may be rapidly released from the food matrix during digestion, or they by fully or partially retained. In solid foods, mastication may facilitate the release of a significant fraction of bioactives. Indeed, an increase in carotenoid bioaccessibility has been reported after mastication of mangoes [31]. However this release is not always complete and there may still be a significant part of bioactive compounds trapped in the food matrix. Moreover, in the case of liquid products (such as tomato juice or orange juice) the passage through the mouth is very fast and therefore the liberation of the bioactives from the plant tissue is very low. In this sense, nanoemulsions may present an effective way to facilitate the release of hydrophobic bioactive compounds into their oil phase during the mouth and stomach phases, that in turn might act as reservoirs of bioactives in the intestine. For instance, there is evidence that the addition of a nanoemulsion to curcumin powder increases the bioaccessibility [32]. The addition of ingredients to an excipient nanoemulsion that favor the dissociation of plant materials may also favor the release of bioactive compounds. For instance, chelating agents might bind calcium from the plant cell wall and increase its permeability and therefore increase the release of bioactive compounds. Also, the incorporation of proteins in the formulation of nanoemulsions might decrease the intestinal pH due to their high buffering capacity [33]. Changes in the pH in the intestinal phase might alter the rate and extent of the breakdown of food matrix and therefore favor the liberation of bioactives entrapped within plant or animal tissue cells.

##### Solubilization in the Intestinal Fluids

The solubilization of hydrophobic bioactive compounds in the aqueous fluids within the GIT is often an important step limiting their bioaccessibility. The co-ingestion of a lipid source has been shown to improve the bioaccessibility of many hydrophobic bioactives by increasing their solubility in GIT fluids [2,34,35]. This fact can be related to a number of factors that favor the solubilization of lipophilic compounds in the GIT fluids [36]. First, the ingestion of a high amount of lipids favors the production of digestion enzymes and bile salts, which increases the solubilization capacity of the mixed micelle phase. Second, the ingestion of foods with high fat levels slows down GIT transit, thereby increasing the time for bioactives to be released from food matrices, solubilized, and absorbed [37]. Third, the release of free fatty acids and monoacylglycerols from the initial triglyceride structure contributes to the formation of mixed micelles that increase the solubilization capacity of the GIT fluids for hydrophobic bioactives. Finally, the use of food-grade surfactants in excipient nanoemulsions can also increase the solubilization capacity of intestinal fluids [38]. This type of knowledge can be used to formulate excipient nanoemulsions that will enhance the solubility of hydrophobic bioactive compounds. Studies have already shown that nanoemulsion-based delivery systems are able to increase the bioaccessibility of carotenoids to a greater extent than conventional emulsions due to their higher digestibility [14]. Moreover, the bioaccessibility of other lipophilic compounds such as coenzyme Q10 can also be enhanced by using emulsion-based delivery systems [39]. The same principle is likely to apply for the design of excipient nanoemulsions. Nanoemulsions have a large exposed lipid surface area, and so they are digested more rapidly and completely in the GIT. Therefore, there are more fatty acids available to form mixed micelles to solubilize the hydrophobic bioactive compounds.

#### 4.3.2. Absorption

**Figure 4:**
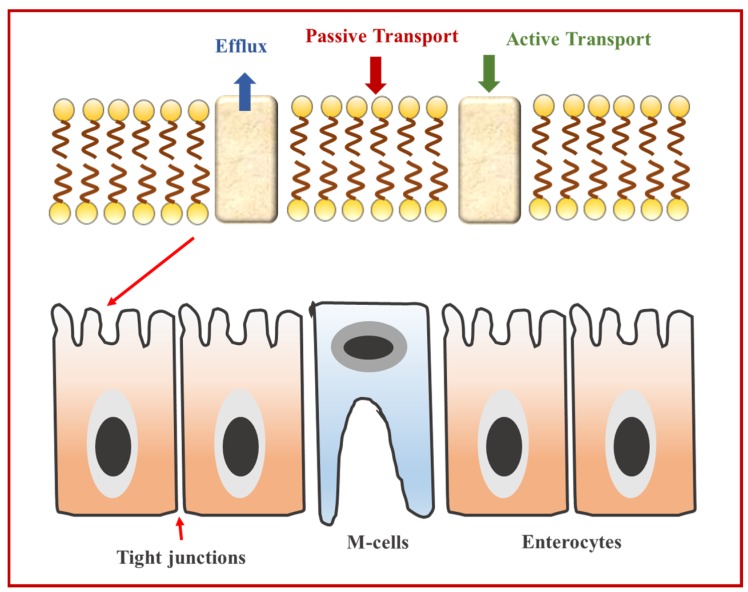
Schematic representation of the mechanisms involved in the absorption of bioactive compounds in the gastrointestinal tract. The absorption of bioactive agents may be limited due to their transport across the epithelium cell through passive, active or efflux mechanisms. Most bioactive compounds are usually absorbed in the upper gastrointestinal tract (GIT) and therefore do not reach the M-cells, but bioactives trapped within indigestible particles or matrices may move further down the GIT and then encounter the M-cells.

After part of the bioactives have been liberated from the food matrix and also solubilized in the intestinal fluids, they have to be transported through the lumen, mucus layer, and epithelium cells lining the small intestine [40] (Figure 4). Therefore, intestinal cell uptake depends on the physical barriers that the bioactive components must overcome. The absorption of a bioactive compound is the amount that is bioaccessible and transferred into the epithelium cells (*m*_BA_) compared to the total amount in the gastrointestinal tract (*m*_Total_) (Equation (3)):

B* = 100 × *m*_BA_/*m*_Total_ (3)

##### Increase in Mucus Layer Transport

The mucus layer is a porous hydrogel layer that coats the enterocytes in the epithelium cells. For the bioactive compound to be transferred to the intestinal cells it has to travel across the mucus layer. If the mixed micelles are larger than the pore size (400 nm) or strongly interact with the mucus layer, then their transport through this layer might be significantly inhibited. For instance, nanoemulsions can be formulated with small molecule surfactants or other stabilizers that help forming small mixed micelles that easily penetrate through the mucus layer [41].

##### Increase in the Permeability of Cell Membranes

Once the bioactive has passed through the mucus layer, it may be incorporated into the intestinal cells by either passive or active transport (Figure 4). The nature and environment of the bioactive compound determines the absorption route taken [42,43,44]. Sufficiently small colloidal particles may be able to travel through the narrow channels separating the epithelial cells known as tight junctions. Therefore, excipient nanoemulsions could be formulated with ingredients that are capable of enlarging the tight junctions [45] such as certain surfactants [46,47]; biopolymers [48,49], mineral ions [50], and chelating agents [51]. Other types of bioactive compounds are transported across the epithelium cell walls by specific or non-specific protein-transporter systems, which consist of one or more proteins embedded in the phospholipid bilayer. Both passive and active transport can be modulated by including excipient ingredients in nanoemulsions such as piperine [52], sucrose monoesters [53], and rhamnolipids [54].

##### Efflux Inhibition

After bioactive compounds are transported into the epithelium cells they may be pumped back into the GIT by certain efflux transporters located in the cell membranes [55,56]. Certain food ingredients might block the efflux transporters, such as quercetin, resveratrol, and piperine that may act as efflux inhibitors for certain pharmaceutical agents [57,58,59,60]. Efflux inhibitors might either block binding sites on the surfaces of efflux proteins, interfere with the energy production mechanism required for efflux or change cell membrane structure thereby altering the conformation and activity of efflux proteins.

#### 4.3.3. Transformation

Many bioactive compounds undergo chemical or enzymatic transformations during their passage though the GIT, which may alter their bioavailability and bioactivity. These transformations may be from an active to an inactive form, or vice versa. For instance, carotenoids are present in different isomer forms in fruits and vegetables, and *cis*-isomers are more bioavailable than *trans*-isomers since the long linear structure of *trans*-isomers are difficult to fit into mixed micelles [61,62]. *Trans*-isomers in foods can undergo isomerization to *cis*-isomers, which might then be absorbed more effectively [63,64]. Consequently, it may be possible to add excipient ingredients to a nanoemulsion to enhance this isomerization reaction. Conversely, certain types of food components under oxidation or other degradation reactions that reduce their biological activity, e.g., carotenoids, ω-3 fatty acids, and curcumin. In these cases, it is possible to add natural or synthetic antioxidants to inhibit their degradation [65]. Therefore, efforts might be put in designing foods that might favor or oppose the chemical or enzymatic conversions of bioactive compounds in the GIT.

### 4.4. Examples of Excipient Nanoemulsions

Recently, a number of studies have demonstrated the potential application of nanoemulsions for enhancing the oral bioavailability of bioactive compounds in natural foods (Table 3). Different strategies have been addressed to enhance the oral bioavailability of bioactive compounds though increasing the bioaccessibility, the absorption, or transformation.

**Table 3:** Examples of potential excipient emulsions or nanoemulsions to be co-ingested with natural food products to enhance the oral bioavailability of bioactive compounds.

Emulsion	Nutraceuticals	Excipient Emulsion Influencing Nutraceuticals Bioaccessibility	Reference
Corn oil emulsions	Curcumin in Powdered Form	The solubility and bioaccessibility of curcumin was significantly improved by incubating and co-ingesting with excipient emulsion.	[32]
Corn oil emulsions	Curcumin in Powdered Form	Emulsifier type and droplet size of exhibited a significant effect on the solubility and bioaccessibility of curcumin.	[66]
Corn oil emulsions	Curcumin in Powdered Form	The bioaccessibility of curcumin depended on oil type and concentration.	[67]
Corn oil, medium chain triglycerides or orange oil emulsions	Carotenoids in yellow peppers	Increased carotenoid bioacessibility from yellow peppers when consumed with corn oil nanoemulsions as excipient emulsions.	[68]
Olive oil emulsions	Carotenoids in Carrot and Tomato suspensions	Adding olive emulsions to carrot and tomato suspensions increased carotenoid uptake in the micellar phase.	[69]
Peanut oil emulsions	Carotenoids in Tomato juice	Lycopene bioaccessibility was dependent on emulsification and emulsifier type	[70]
Various emulsions	Carotenoids in vegetables and salads	Addition of oil to salads and vegetables increased lycopene bioaccessibility depending on fatty acid type	[71,72]

#### 4.4.1. Increase in Bioaccessibility

For powdered nutraceuticals, nanoemulsions have been shown to significantly increase the transfer of the bioactive crystalline form to the oil phase, which is directly related to an increased solubility in the GIT fluids. For instance, Zou and co-workers [32,66] reported an increased *in vitro* bioaccessibility of powdered curcumin after mixing with excipient nanoemulsions, which simulated a salad dressing (incubation at 30 °C) or a cooking sauce (incubation at 100 °C). They found a higher transfer of curcumin powder to the oil droplets when nanoemulsions were incubated at 100 °C compared to 30 °C. In the case of bioactive compounds in plant-based products, lipid droplets in nanoemulsions lipid droplets may act as a non-polar solvent that facilitates the liberation of the hydrophobic bioactives from their original location in plant tissues [70]. In fact, the results from several studies with different vegetables are consistent and support this statement. For example, an increase in carotenoid bioaccessibility from yellow peppers has been found after being mixed with excipient nanoemulsions [68]. Moreover, the solubilization of carotenoids in the micelle fraction after *in vitro* digestion of carrot and tomato suspensions was significantly increased after being co-ingested with olive oil emulsions.

There are several factors that influence the ability of excipient nanoemulsions to enhance the bioaccessibility of bioactive compounds, such as the lipid amount, size and composition. For instance, it is known that the higher the oil concentration in the formulation of bioactive-loaded nanoemulsions, the higher the bioaccessibility [29]. This behavior also occurs with excipient nanoemulsions or emulsions consumed with bioactive-rich foods. It has been reported that full-fat salad dressings enhance the oral bioavailability of bioactive compounds to a higher extent than low-fat dressings [17]. Also, the oil composition influences the solubilization of hydrophilic nutraceuticals in the intestinal juices, since the free fatty acids will form the non-polar domains of the mixed micelle phase. It is known that nanoemulsions formulated with long chain triglycerides show an enhanced bioaccessibility of hydrophobic bioactives than medium or short chain triglycerides, which is attributed to a larger micelle structure able to accommodate the hydrophobic bioactives in their core [28,29]. This phenomenon is important when designing excipient nanoemulsions since the same principle would apply for nanoemulsions that are co-ingested with natural foods, as the bioactive compounds might already be transferred into the oil phase prior to digestion. Moreover, the nanoemulsion droplet size is an important factor that determines the rate of lipid digestion rate and mixed micelle formation. The smaller the droplet size, the higher the lipid hydrolysis rate and in turn the higher the bioaccessibility [14,73]. This behavior has been also detected when designing excipient nanoemulsions [66].

The ability of nanoemulsions to enhance the solubilization of lipophilic bioactive compounds in intestinal juices has been demonstrated for numerous types of hydrophobic bioactive agents. Nevertheless, the overall bioavailability of the solubilized fraction has been studied far less frequently. Even so, some studies have shown that hydrophobic bioactive compounds encapsulated within nanoemulsions have a higher bioavailability than those encapsulated within conventional emulsions [74]. On the other hand, to the best of the authors’ knowledge, there have been no reports on the improvement of the bioavailability of bioactive compounds using excipient nanoemulsions to date. Therefore, it is important to elucidate the potential efficacy of excipient nanoemulsions using animal and human feeding studies.

#### 4.4.2. Changes in Absorption and Transformation

Strategies developed to enhance the absorption or transformation of bioactive compounds in the gastrointestinal tract using excipient nanoemulsions is currently scarce, but it is certainly an important topic that should be studied more to improve the ability of excipient nanoemulsions to boost the oral bioavailability of bioactive compounds. For instance, it is known that oil type influences the absorption of bioactives in the gastrointestinal tract as they influence the nature of the chylomicrons formed [75,76]. Oleic acid has also been shown to be an effective inhibitor of efflux mechanisms [77], which may help increase the absorption of certain bioactive compounds. Moreover, the intestinal absorption of hydrophobic bioactives has been enhanced by their ingestion along with piperine from black pepper, which alters the epithelium cell transport mechanisms [78]. Regarding the molecular and structural transformation of bioactive compounds during their passage though the intestinal tract, efforts have been made to design structures to protect them from oxidation and degradation. For instance, the presence of chelating agents, such as EDTA, or antioxidants, such as tocopherol, has shown to decrease the degradation rate of astaxanthin [79]. This would also be valid for the design of excipient nanoemulsions, which might contain those ingredients either in the aqueous or lipid phases and would protect the natural bioactive compounds in foods. The type of antioxidant used to formulate excipient nanoemulsions might be specifically selected depending on the type of bioactive compound to be protected. For instance, lipophilic bioactive compounds such as tocopherols can be included in the lipid phase to prevent degradation of carotenoids. On the other hand, chelating agents (such as EDTA) can be incorporated into the aqueous phase to bind with and inactivate pro-oxidant metal ions (such as iron). Thus, even though the current knowledge regarding the exact mechanism about how to improve the oral bioavailability of different natural bioactives in food is still to be fully elucidated, the available information clearly highlights the greater potential of nanoemulsions as excipient foods.

## 5. Conclusions

In this review article we focused on describing the potential advantages of using nanoemulsions as excipient foods to increase the oral bioavailability of naturally occurring bioactive compounds. It is important to understand the fate of health-related food components in the gastrointestinal tract so as to formulate nanoemulsions with specific structures and compositions known to boost bioavailability and maintain bioactivity. Excipient nanoemulsions may be used to enhance the bioaccessibility and absorption of bioactive compounds in the gastrointestinal tract, as well as controlling their molecular form to ensure they are in the most bioactive state. In certain applications, nanoemulsions have advantages over conventional emulsions because their small droplet size and higher surface area leads to rapid digestion and mixed micelle formation. The co-ingestion of nanoemulsions with foods, supplements or drugs may be a useful method to optimize the uptake of hydrophobic bioactives. Nevertheless, there is still a need to unravel the complex physicochemical and physiological processes involved when excipient nanoemulsions and foods pass through the GIT. This information might be highly valuable for the food and pharmaceutical sector to optimize the bioavailability of bioactive compounds.
